# Physical activity, body weight, and pancreatic cancer mortality

**DOI:** 10.1038/sj.bjc.6600782

**Published:** 2003-03-04

**Authors:** I-M Lee, H D Sesso, Y Oguma, R S Paffenbarger

**Affiliations:** 1 Department of Medicine, Division of Preventive Medicine, Brigham and Women's Hospital and Harvard Medical School, 900 Commonwealth Avenue East, Boston, MA 02215, USA; 2 Department of Epidemiology, Harvard School of Public Health, 677 Huntington Avenue, Boston, MA 02115, USA; 3 Sports Medicine Research Center, Keio University, 4-1-1 Hiyoshi, Kohoku-ku, Yokohama, Kanagawa 223-0061, Japan; 4 Department of Health Research and Policy, Division of Epidemiology, HRP Redwood Building T213B, Stanford, CA 94305, USA

**Keywords:** body weight, epidemiology, exercise, pancreatic cancer, physical activity

## Abstract

In a study of 32 687 subjects with data on physical activity and body mass index (BMI) collected serially over time, we examined associations with pancreatic cancer mortality (*n*=212). Despite plausible biologic mechanisms, neither physical activity (multivariate relative risks for increasing levels: 1.00, 0.98, 0.92, and 1.31, respectively) nor BMI (corresponding findings: 1.00, 0.84, 1.08, and 0.99, respectively) significantly predicted pancreatic cancer mortality.

Pancreatic cancer is a rapidly fatal cancer with 5-year survival of about 4% for all tumours (Ries *et al*, 2002), and <1% for nonresectable tumours (Bramhall *et al*, 1995). In the United Kingdom, this cancer does not rank in the top 10 for newly diagnosed cases, yet, it is the sixth most common cancer death (CancerStats, June 2002; September 2002). Owing to the grim prognosis, prevention of pancreatic cancer is important. Unfortunately, there are few established risk factors, the exception being cigarette smoking (IARC, 1986). Experiments using a hamster model have suggested that insulin and insulin resistance may play a role in the aetiology of pancreatic cancer (Schneider *et al*, 2001). This is supported by human studies, with a meta-analysis of 20 studies concluding that diabetes mellitus is associated with increased risk (Everhart and Wright, 1995). Additionally, a recent study reported that diets rich in foods that raise postprandial glucose levels increase the risk of pancreatic cancer (Michaud *et al*, 2002).

Physical inactivity and overweight are associated with abnormal glucose metabolism, including insulin resistance, hyperinsulinaemia, impaired glucose tolerance, and type II diabetes (Albu and Pi-Sunyer, 1998; Kelley and Goodpaster, 2001). Owing to these associations, we hypothesised that physical activity and a lean body weight would decrease the risk of pancreatic cancer. Few data are available, providing the motivation for this study.

## MATERIALS AND METHODS

### Subjects

The College Alumni Health Study is an ongoing study of physical activity and health among men and women in the United States, initiated in 1962. Subjects were recruited from men matriculating at Harvard University as undergraduates between 1916 and 1950, and men and women matriculating at the University of Pennsylvania as undergraduates and graduates between 1928 and 1940. The first health survey was mailed to Harvard University alumni in either 1962 or 1966, and University of Pennsylvania alumni in 1962. We have since obtained updated health information via mailed surveys at periodic intervals.

In 1962 or 1966, 21 582 Harvard men and 14 342 University of Pennsylvania alumni returned their initial health survey. For the present study, we excluded subjects reporting cancer at baseline (*n*=601), and those with missing information on age or sex (*n*=74) and physical activity, body weight, or height (*n*=1645). Of the remaining 33 604 subjects, we successfully followed 32 687 (including 2302 women) for pancreatic cancer mortality; these represent the subjects for this study.

### Assessment of physical activity and body weight

Harvard alumni provided information on physical activity and body weight at baseline in 1962 or 1966 and updated this in 1977, 1988, and 1993. University of Pennsylvania alumni provided the same at baseline in 1962, updating this in 1980 and 1993. For physical activity, we asked about daily walking and stair climbing, and sports and recreational activities undertaken in the past week, as well as the frequency and duration of participation (Lee *et al*, 1992). This assessment of physical activity has been shown to be reliable and valid (Albanes *et al*, 1990; Washburn *et al*, 1991; Ainsworth *et al*, 1993b). For example, the test–retest correlation coefficient over 1 month was 0.72, while estimates of energy expenditure from questionnaires compared with physical activity records yielded a correlation coefficient of 0.65 (Ainsworth *et al*, 1993b).

### Assessment of other characteristics

We also obtained information on cigarette, cigar, and pipe smoking and physician-diagnosed diabetes mellitus at baseline. These data (except cigar and pipe smoking) were updated in 1977, 1988, and 1993 for Harvard alumni, and 1980 and 1993 for University of Pennsylvania alumni. Information on alcohol, coffee, and tea consumption was obtained in 1977 and 1988 for the former subjects, and 1980 for the latter.

### Ascertainment of pancreatic cancer mortality

The Alumni Office of both universities maintains records of deceased alumni. Using these records, we obtained copies of death certificates to ascertain the underlying and contributing causes of mortality.

### Data analyses

At each assessment, we estimated total energy expenditure from walking, stair climbing, and participation in sports and recreation (Lee and Paffenbarger, 2000). We categorised subjects into approximate fourths according to the baseline distribution: <2100, 2100–4199, 4200–10 499, and ⩾10 500 kJ week^−1^. For the different components of physical activity, we categorised subjects into approximate fourths of distance walked and stories climbed (both classified as in Table 2

**Table 2:** Relative risks (RR) and 95% confidence intervals (CI) of pancreatic cancer, according to physical activity^a^ and body mass index^a^

**Characteristic**	**No. of cases^b^**	**No. of subjects^b^**	**Age- and sex-adjusted RR (95% CI)**	**Multivariate RR^c^ (95% CI)**
*Physical activity,^d^* kJ week^−1^				
<2100	56	8667	1.00 (referent)	1.00 (referent)
2100–4199	46	7168	0.98 (0.65–1.47)	0.98 (0.65–1.49)
4200–10 499	50	9514	0.93 (0.63–1.35)	0.92 (0.62–1.35)
⩾10 500	60	7338	1.27 (0.87–1.84)	1.31 (0.69–1.92)
			*P*, trend=0.28	*P*, trend=0.24
*Distance walked, km week*^−*1*^				
<5	63	9618	1.00 (referent)	1.00 (referent)
5–<10	47	7165	0.84 (0.56–1.24)	0.81 (0.54–1.23)
10–<20	78	11 932	0.90 (0.65–1.26)	0.89 (0.63–1.25)
⩾20	24	3972	0.99 (0.66–1.50)	0.99 (0.64–1.51)
			*P*, trend=0.85	*P*, trend=0.82
*Stories climbed, week*^−*1*^				
<10	45	7419	1.00 (referent)	1.00 (referent)
10–<20	52	7047	1.14 (0.77–1.68)	1.12 (0.75–1.68)
20–<35	34	6403	0.78 (0.50–1.22)	0.77 (0.49–1.22)
⩾35	81	11 818	1.20 (0.85–1.69)	1.19 (0.83–1.71)
			*P*, trend=0.51	*P*, trend=0.54
*Participation in light activities (<4 METs)*^e^				
No	186	28556	1.00 (referent)	1.00 (referent)
Yes	26	4131	1.14 (0.78–1.67)	1.11 (0.74–1.64)
				
*Participation in moderate activities (4–<6 METs)*^e^				
No	176	27 622	1.00 (referent)	1.00 (referent)
Yes	36	5065	1.33 (0.99–1.79)	1.37 (1.00–1.87)
				
*Participation in vigorous activities (⩾6 METs)*^'^^e^				
No	177	25 819	1.00 (referent)	1.00 (referent)
Yes	35	6868	0.82 (0.59–1.15)	0.86 (0.61–1.21)
				
*Body mass index, kg m*^−*2*^				
<22.5	48	7612	1.00 (referent)	1.00 (referent)
22.5–<25.0	76	12 637	0.84 (0.59–1.21)	0.84 (0.58–1.22)
25.0–<27.5	66	9100	1.05 (0.73–1.52)	1.08 (0.74–1.57)
⩾27.5	22	3 338	1.00 (0.62–1.61)	0.99 (0.60–1.62)
			*P*, trend=0.67	*P*, trend=0.66

a Assessed in 1962 or 1966 and updated in 1977, 1988, and 1993 for Harvard University alumni; assessed in 1962 and updated in 1980 and 1993 for University of Pennsylvania alumni.

b Number of cases and subjects are distributed according to baseline values.

c Adjusted for age (single years), sex, cigarette smoking (never; past; current smoker of ⩽1 pack day^−1^, >1 pack day^−1^, or unknown amount), and diabetes mellitus (no *vs* yes); additionally, each component of physical activity (distance walked, stories climbed, participation in light, moderate, or vigorous activities) also is adjusted for the remaining components (categorized as shown in the table). Cigarette smoking, diabetes mellitus, and physical activity components were assessed as in footnote (a) above.

d Estimated from walking, climbing stairs, and participating in sports and recreational activities.

e METs, multiples of resting metabolic rate.

), and dichotomised them into not participating or participating in light-, moderate-, and vigorous-intensity activities (Ainsworth *et al*, 1993a; details provided in Table 2). For body weight, we calculated body mass index (BMI; weight height^−2^) at each assessment and divided subjects into approximate fourths using the baseline distribution: <22.5, 22.5 – <25, 25 – <27.5, and ⩾27.5 kg m^−2^.

We used proportional hazards regression with time-dependent covariates (SAS, 1999) to estimate the hazard ratios (relative risks) of pancreatic cancer mortality associated with physical activity and BMI. Initially, we adjusted for age and sex. In age- and sex-adjusted models, cigarette smoking and diabetes mellitus, but not cigar or pipe smoking, alcohol, coffee, or tea intake, significantly predicted mortality from pancreatic cancer. Therefore, to control for these predictors, we additionally included cigarette smoking and diabetes mellitus (classified as in Table 2) in multivariate models. When we examined the association of the each component of physical activity – walking, climbing stairs, and participating in light, moderate, or vigorous activities – with the risk of pancreatic cancer, we further controlled for the other components.

## RESULTS

Among the 32 687 subjects, the mean age at baseline was 47. 1 years and 93% were male. During follow-up through 1995, 212 persons died from pancreatic cancer. Table 1

**Table 1:** Characteristics of subjects at baseline, 1962 or 1966, according to physical activity^a^

	**Physical activity, kJ week^−1^**			
	**<2100**	**2100–4199**	**4200–10 499**	**⩾10 500**
**Characteristic**	**(*n*=8667)**	**(*n*=7168)**	**(*n*=9514)**	**(*n*=7338)**
Mean age (s.d.), year	47.7 (8.7)	47.2 (8.7)	46.6 (8.8)	46.8 (8.7)
% male	86.7	92.2	95.5	97.8
Mean distance walked (s.d.), km week^−1^	2.9 (2.1)	8.7 (3.2)	13.3 (9.1)	17.9 (16.7)
Mean stories climbed (s.d.), week^−1^	18.7 (16.7)	29.7 (25.0)	32.6 (34.3)	35.1 (35.9)
*% participating in:*				
Light activities (<4 METs)^b^	0.4	3.8	17.1	29.9
Moderate activities (4–<6 METs)	0.6	4.7	21.2	36.3
Vigorous activities (⩾6 METs)	0.6	6.0	28.7	49.8
*% body mass index, kg m*^−*2*^				
<22.5	24.6	24.4	24.1	19.7
22.5<25.0	35.3	36.9	41.0	41.3
25.0<27.5	27.9	28.1	26.2	29.7
⩾27.5	12.2	10.6	8.7	9.4
% smoking cigarettes	48.0	48.4	48.1	48.0
% smoking cigars	15.9	16.9	18.0	20.0
% smoking pipes	19.2	21.3	22.9	24.0
% with diabetes mellitus	2.3	2.0	1.5	1.4
% consuming alcohol daily^c^	52.5	56.2	58.9	64.6
% drinking >2 cups of coffee day^−1^ c	37.6	37.9	37.9	37.6
% drinking >2 cups of tea day^−1^ c	9.0	9.2	9.4	9.2

a Estimated from walking, climbing stairs, and participating in sports and recreational activities.

b METs, multiples of resting metabolic rate.

c This information was not available at baseline, but in 1977 for Harvard alumni and 1980 for University of Pennsylvania alumni.

shows the baseline characteristics of subjects according to physical activity level. The more active subjects tended to be younger, male and, as expected, participated more in all the different activity components. Active individuals tended to be less overweight and were more likely to smoke cigars or pipes; however, the proportions smoking cigarettes were similar among the different activity groups. These smoking patterns were observed at a time when the health hazards of smoking were not well known. Active subjects had a lower prevalence of diabetes mellitus and were more likely to consume alcohol, but did not differ from the less active in coffee or tea consumption.

In age- and sex-adjusted analysis, higher levels of energy expenditure did not predict lower risk of pancreatic cancer mortality (*P*, trend=0.28; Table 2). The most active subjects, expending ⩾10 500 kJ week^−1^, had a relative risk of 1.27 (95% confidence interval, 0.87–1.84) compared with the least active expending lt;2100 kJ week^−1^. When we examined the separate components of physical activity (walking, climbing stairs, participation in light, moderate, or vigorous activities), none showed a significant association with pancreatic cancer mortality. Further adjustment for cigarette smoking and diabetes mellitus, as well as participation in the other components, did not materially alter these findings.

For BMI, there was also no significant relation with the risk of pancreatic cancer mortality in age- and sex-adjusted models (*P*, trend=0.67; Table 2). The heaviest subjects, with BMI⩾27.5 kg m^−2^, had a relative risk of 1.00 (95% confidence interval, 0.62–1.61) compared with the leanest, with BMI <22.5 kg m^−2^. Again, further adjustment for cigarette smoking and diabetes mellitus did not change the results.

Finally, we investigated the joint effect of physical activity and BMI on pancreatic cancer risk. We classified subjects into four groups according to whether they met the guidelines for physical activity (Pate *et al*, 1995) and body weight (WHO, 1995) at baseline: sedentary (<4200 kJ week^−1^) and overweight (BMI ⩾25 kg m^−2^), sedentary and normal weight (BMI <25 kg m^−2^), active (⩾4200 kJ week^−1^) and overweight, and active and normal weight. The multivariate relative risks of pancreatic cancer were 1.00 (referent), 1.04 (95% confidence interval, 0.81–1.92), 1.25 (0.81–1.92), and 0.97 (0.65–1.46), respectively.

## DISCUSSION

This large study of men and women does not support the hypothesis that higher levels of physical activity and leaner body weight decrease the risk of pancreatic cancer. Individuals who satisfied recommendations for physical activity (Pate *et al*, 1995) and/or a healthy BMI (WHO, 1995) did not experience lower risk of pancreatic cancer than their less active and overweight peers.

Biologically, it appears plausible for physical activity and lean body weight to decrease pancreatic cancer risk. Physical inactivity and overweight are associated with abnormal glucose metabolism, with 90% of type II diabetics in the US being obese (Albu and Pi-Sunyer, 1998). On the other hand, physical activity improves insulin action in overweight and obese individuals, even if body weight and composition are unchanged (Kelley and Goodpaster, 1999). An abnormal insulin and glucose profile is related to increased risk of pancreatic cancer. Hamsters fed a high-fat diet experience peripheral insulin resistance and develop pancreatic cancers when exposed to a pancreatic carcinogen (Schneider *et al*, 2001). However, if treated with metformin, which improves insulin sensitivity and can lead to lower insulin levels, the incidence of pancreatic cancer is very much reduced. Human pancreatic cancer cells possess insulin receptors, and dose-dependent increases in cell proliferation are observed in response to insulin (Fisher *et al*, 1996). In humans, higher plasma glucose levels after an oral glucose load is predictive of pancreatic cancer mortality (Gapstur *et al*, 2000), and a diagnosis of diabetes mellitus is associated with increased risk (Everhart and Wright, 1995).

While the above data suggest that physical activity and body weight may have a role in the aetiology of pancreatic cancer, the findings from epidemiologic studies have been sparse and inconsistent. Three small studies reported no significant association between physical activity and risk (Paffenbarger *et al*, 1987; Garfinkel and Stellman, 1988; Waterbor *et al*, 1988), as did a large study with 409 pancreatic cancer deaths (Brownson *et al*, 1991). In contrast, a prospective study with 350 incident cases observed about a 30–40% reduction in risk among the most active subjects, especially those overweight (Michaud *et al*, 2001). It is unclear why the present findings differ. Our *a priori* calculations had estimated reasonable power to detect a 40% reduction in pancreatic cancer risk.

Previous studies of body weight and pancreatic cancer also have yielded inconsistent findings, with case–control studies being more likely to report no association (Howe *et al*, 1992; Lyon *et al*, 1993; Coughlin *et al*, 2000; Michaud *et al*, 2001). A possible explanation for the discrepancy may be that because of the high mortality from pancreatic cancer, case–control studies are limited by the information obtained from next of kin. However, this study that obtained information on weight prospectively and directly from subjects also did not observe any association with BMI.

Strengths of the present study include its large size and detailed physical activity information. As discussed, the prospective nature of this study limits potential bias from a high fatality rate among pancreatic cancer patients. Limitations include the self-reported information on physical activity and body weight. However, our physical activity assessment is reliable and valid for large population studies (Albanes *et al*, 1990; Washburn *et al*, 1991; Ainsworth *et al*, 1993b). Self-reported body weight also correlates well with measured weights in a well-educated population (Rimm *et al*, 1990). We were limited in our ability to examine the sexes separately because of the small number of cases in women (*n*=5). Finally, we ascertained mortality not incidence, but this should not represent a major limitation since, with its high fatality rate, pancreatic cancer mortality would closely reflect incidence.

In conclusion, the data from this large study do not support the biologically plausible hypothesis that physical activity and lean body weight to reduce the risk of pancreatic cancer.
